# Preclinical Murine Models for Lung Cancer: Clinical Trial Applications

**DOI:** 10.1155/2015/621324

**Published:** 2015-05-03

**Authors:** Amelia Kellar, Cay Egan, Don Morris

**Affiliations:** Departments of Oncology/Medicine, University of Calgary, 1331 29 Street NW, Calgary, AB, Canada T2N 4N2

## Abstract

Murine models for the study of lung cancer have historically been the backbone of preliminary preclinical data to support early human clinical trials. However, the availability of multiple experimental systems leads to debate concerning which model, if any, is best suited for a particular therapeutic strategy. It is imperative that these models accurately predict clinical benefit of therapy. This review provides an overview of the current murine models used to study lung cancer and the advantages and limitations of each model, as well as a retrospective evaluation of the uses of each model with respect to accuracy in predicting clinical benefit of therapy. A better understanding of murine models and their uses, as well as their limitations may aid future research concerning the development and implementation of new targeted therapies and chemotherapeutic agents for lung cancer.

## 1. Introduction

Lung cancer is the leading cause of cancer mortality worldwide [1]. It is estimated that approximately 228,190 people were diagnosed with lung cancer in 2013, resulting in approximately 159,480 deaths in the United States [2]. Current chemotherapies prove to be only marginally effective in extending overall survival as five-year survival for anyone diagnosed with cancer of the lung or bronchus is about 16% [2]. The development and implementation of new, targeted agents may be aided by the availability of universally applicable experimental murine models for testing novel therapeutics. In order to generate and evaluate novel therapies for lung cancer, advanced preclinical models ideally should accurately mimic lung cancer progression, invasion, and metastasis as well as predicting clinical benefit of therapy for all types of lung cancer. A wide variety of murine model systems have been developed with the aim of not only evaluating novel therapeutics, but also examining the mechanisms underlying transformation, invasion and metastasis in human tumours with a view to better study prevention and screening as well as diagnostic and treatment strategies. This review will introduce the frequent mutations found in lung cancer patients and how these mutations have been incorporated into preclinical models to accurately evaluate novel therapies for lung cancer. Characteristics of each model system as well as the advantages and disadvantages will be described. Relevant models will then be discussed with regard to how accurate each murine model is in successfully predicting outcome of therapy in clinical trials.

## 2. Mutations Associated with Lung Cancer Development

A better understanding of the most frequent driving mutations in lung cancer will aid in the progression towards more personalized therapy. Molecular markers have been identified that provide the basis for targeted therapies for lung cancer. Current prognostic molecular pathways for lung cancer include EGFR, K-Ras, p53, and EML4-ALK [3–9].

EGFR regulates a myriad of cell functions such as proliferation, angiogenesis, and apoptosis [6]. The most common EGFR activating mutations are in-frame deletions in exon 19 or point mutations in codon 858 in exon 21 [10]. Targeted therapies in the form of EGFR tyrosine kinase inhibitors, such as erlotinib and gefitinib as well as monoclonal antibodies against EGFR such as cetuximab, have been employed as treatments for the disease. EGFR-targeted therapies have proven to be effective in both first and second-line of treatment for patients with EGFR mutations [11].

Mutations in the K-Ras gene are present in approximately 30 percent of adenocarcinomas and are generally associated with a poor prognosis [12]. The K-Ras oncogene encodes a family of membrane-bound guanosine triphosphate- (GTP-) binding proteins that are involved in cell proliferation, migration, and apoptosis. The most common K-Ras mutations are in the form of point mutations on exons 12 and 13, typically resulting in constitutive activation of RAS [13]. Interestingly, cases of NSCLC exhibiting K-Ras mutations are predominately resistant to the EGFR inhibitors, erlotinib, and gefitinib [14].

In addition to K-Ras, p53 is a well-established predictive and prognostic marker for NSCLC. Loss of the tumour suppressor gene, p53, leads to mitotic abnormalities during cellular development resulting in highly proliferative cells [15]. Transversions along the p53 gene are found in almost all human lung cancer tissues and have implicated p53 as a key molecular marker for lung cancer [16]. A comprehensive meta-analysis of the role of p53 as a prognostic factor for lung cancer survival revealed that mutated or inactive p53 was shown to be associated with a poor survival [17].

It has recently been reported that echinoderm microtubule-associated protein-like 4 (EML4) and anaplastic lymphoma kinase (ALK) gene fusions are present in approximately 3% of patients with NSCLC and that EML4 and ALK amplifications may play a role in NSCLC transformation [9]. NSCLC and SCLC have also been associated with mutations in the PI3K-Akt-mTOR pathway, LKB1, TITF1, beta-tubulin, ERCC1, and RRM1 [18–22].

## 3. Xenograft,* Ex Vivo*, and Orthotopic Models

For the purpose of this review, murine models can be divided into the following groups: xenograft, transgenic, syngeneic, and spontaneous model systems. Xenograft models require the injection of human cancer cells into immunocompromised mice, either subcutaneously, orthotopically, or systemically. Immunocompromised mice such as athymic nude and severe-compromised immunodeficient (SCID) mice are frequently utilized as implanted human cells are likely to be rejected by the host immune system in an immunocompetent system. Once implanted, cells require a growth period of one to eight weeks depending on cell type and the number of cells injected. Xenograft models are primarily used to examine tumour response to therapy* in vivo* prior to translation into clinical trials. Cell lines and current xenograft models for the study of lung cancer are summarized in Table 1.

Cancer cell lines vary in optimal cell number required for implantation, ranging from 1 × 10^6^ − 1 × 10^7^ cells/injection site. Both the average number of tumours that engraft (tumour take) and the average time to palpable tumours are dependent on the number of cells implanted, growth characteristics of each cell-line such as doubling time, cell-size, density, morphology, and the use of growth factors such as matrigel. Cell lines commonly used to model adenocarcinoma are A549, H1975, HCC4006 and HCC827 [26, 28, 43], representing a spectrum of K-Ras and EGFR mutations. Current xenograft models for adenocarcinoma demonstrate an average tumour take of 50–100%, with the A549 cell-line as the most likely to engraft [26, 28, 37, 38]. The tumour take as well as murine strain used for implantation are given in Table 1. Typically, these models require two to eight weeks following cell implantation in order to observe tumour growth substantial enough for evaluating drug efficacy.

Cell-lines that are commonly used to model carcinoma [27, 29, 32], large-cell carcinoma [31, 33–36], and squamous-cell carcinoma [36] in xenograft models include NCI-H1299, NCI-H460 and NCI-H226, respectively. The NCI-H460 cell-line has proven to be an advantageous model system as it requires small implantation cell numbers and limited growth time and has been shown to have a 100 percent tumour take when injected into the hind flank of CD-1 athymic nude mice [28, 36]. Both the NCI-H1299 and the NCI-H226 cell-lines are slightly more limited in their experimental uses as they have only a 45–100 percent tumour take and require at least four weeks to reach optimal tumour size in order to begin treatment [27, 29, 36, 65]. Models for SCLC are generally limited, however, the NCI-H69 and DMS-53 cell-lines are the most widely used for xenograft studies but can be problematic as they characteristically grow in suspension, resulting in difficulty in obtaining an accurate cell count prior to implantation [23–25]. These characteristics may contribute to a highly variable tumour take and growth rates of these models.

In addition to traditional xenograft models,* ex vivo* models can be used in which tumours are surgically removed from patients and tumour cells are grafted into the immunocompromised murine system either subcutaneously or orthotopically. These models are ideal for personalized therapy and provide relatively quick data concerning the most beneficial therapies for each patient [89–91]. In the study conducted by Dong et al. [91], thirty-two untreated samples of NSCLC were engrafted into the renal capsules of nonobese diabetic/SCID mice. Tumour growth was evaluated in response to cisplatin, docetaxel, and gemcitabine.* Ex vivo* tumour take was 90 percent and results were obtained over six to eight weeks. As a result, therapy regimens for each patient were tailored according to observed tumour response in the xenograft models. A good correlation was found between recurrence or metastasis in patients and the non-responsiveness of their tumour xenografts in mice.

There is ample evidence that growth properties of tumour cells are altered by specific genes whose expression is dependent on interactions within the tumour microenvironment. Therefore, it is vital that tumour microenvironment be accurately mirrored in murine models used to evaluate drug therapies. Orthotopic models provide a reliable representation of tumour environment as cells are implanted directly into the organ in which the disease originates. Current orthotopic models are reviewed in Table 1. The most practical orthotopic model involves endobronchial inoculation of the A549 or H460 cell-lines into athymic NCr-nu/nu mice [31]. The procedure results in a postsurgery mortality rate of less than 5 percent. The rate of tumour engraftment is 90 percent and tumour growth is monitored through high-resolution chest roentgenography or bioluminescence via transfection of luciferase containing constructs [92].

Xenograft models for lung cancer have advantages and disadvantages in comparison with classic transgenic and conditional murine models. Firstly, xenograft models utilize human tumour tissue, perhaps accurately representing the complexities of human tumours* in vivo*. Unlike their genetically engineered counterparts, xenograft models can be used to design individualized molecular therapy. In a study by John et al. [93], the ability of tumour fragments from patients undergoing curative surgery to engraft into primary tumour xenografts was found to be predictive of risk of disease recurrence. These findings, in conjunction with other* ex vivo* xenograft findings show that xenograft models are a useful evaluative tool for targeted molecular therapy and predicting patient outcome [91]. Xenograft models are also ideal for examining multitherapy approaches* in vivo*. Much chemotherapy is approved on the basis of a combination therapy regimen with other preexisting interventions, therefore, pre-clinical xenograft models are used to evaluate efficacy of these drug combinations prior to clinical trials [32, 76, 81, 94]. Orthotopic xenograft models provide the very valuable advantage of accurate representation of the tumour microenvironment in evaluating drug therapies. This allows for a reliable prediction of toxicity, and understanding of microenvironment-dependent responses to selected therapies.

Despite the advantages of using xenograft models in preclinical studies, there are also many limitations to these models that must be addressed. Immunocompromised mice must be used for xenograft models in order to combat the effects of the healthy immune system response against foreign cells. Syngeneic models, which will be discussed shortly, are alternative model systems used to combat this issue. Alternatively, orthotopic models combat the issue of inaccurate representation of tumour microenvironment as cells are implanted directly into the bronchi; however, once growth commences, it is more difficult to quantify than in the traditional xenograft model. Current orthotopic models used to study lung cancer are shown in Table 1. Despite the benefits of orthotopic systems, they can also be quite time consuming and challenging to replicate as cell inoculations are typically conducted endobronchially, requiring skillful precision and practice. These disadvantages may account for the lack of robust orthotopic models for lung cancer, lending to the preference towards the traditional hind flank xenograft model.

## 4. Syngeneic Models

Syngeneic murine models entail the injection of immunologically compatible cancer cells into immunocompetent mice. The availability of syngeneic models to study lung cancer is very limited. The only reproducible syngeneic model for lung cancer to date is the Lewis lung carcinoma (LLC) model. LLC is a cell line established from the lung of a C57BL mouse bearing a tumour resulting from the implantation of primary Lewis lung carcinoma. The cell line is highly tumourigenic and is primarily used to model metastasis as well as evaluate the efficacy of chemotherapeutic agents* in vivo* [95]. For example, the LLC model was a successful preclinical model for Navelbine evaluation* in vivo*, prior to its implementation in clinical trials [73, 74]. The LLC cell-line is typically injected orthotopically into the peritoneal cavity of C57B6 mice at 1 × 10^7^ cells per mouse and within two weeks of incubation, tumours reach 2.2 ± 0.4 mm [40, 73]. Preclinical models for evaluation of chemotherapeutic agents are shown in Table 3.

The advantage of the LLC model is that implanted cells are immunologically compatible with the murine system, unlike the widely used xenograft models in which human cells are implanted into mouse tissue. As a result, LLC models can be created on an immunocompetent murine background, such as C57BL, and true immune and toxicity responses can be evaluated with respect to targeted therapies and tumour growth. In addition, because the LLC model can be both syngeneic and orthotopic, tumour microenvironment can be accurately depicted in the animal model. Despite its superiority as an animal model for lung cancer, the LLC model is associated with several limitations. As a syngeneic model, responses evaluated in a complete murine system may not be transferable to human conditions. As an orthotopic model, the LLC model can also result in difficulties in quantifying tumour growth without advanced imaging equipment and as such, it can be quite expensive, time-consuming, and difficult to reproduce.

## 5. Transgenic and Conditional Transgenic Models

Genetically engineered models (GEM) are used to induce spontaneous neoplastic growth via transgenic, conditional, or drug-induced mechanisms. Transgenic mice are created by microinjection of DNA into the pronucleus of zygotes and injection of embryonic stem cells into blastocysts to produce the desired loss or gain of function mutations. Transgenic mouse models for lung cancer may be general, where tumours arise in lung and in organs other than the lung or specific, where the lung alone is the target of the transgene. The latter models are more useful, as the frequency of the development of lung cancer is often higher and the pathology of the disease is not complicated by tumours at other sites. The DNA construct for the transgene is created by linking a lung-specific promoter to the coding region of a target gene [96]. Transgenic mice are ideal for examining the role of genetic abnormalities in tumour initiation and progression. The current transgenic models that are used to study lung cancer are shown in Table 2.

One of the first viral oncogenes to be targeted to the lung was Simian virus T antigen (TAg). Tag binds to and inactivates p53 and pRB, both of which have been reported to be mutated or functionally altered in lung cancer [97]. Through the use of the lung specific promoters Clara cell secretory protein (CCSP), also known as uteroglobin promoter, and alveolar type II surfactant protein C (SP-C), these transgenes resulted in the development of adenocarcinoma in a murine model [53, 54]. The mice developed multifocal bronchioalviolar neoplasias very rapidly and often died before four months of age, making investigation of the early events in carcinogenesis difficult. An alternative model for pulmonary adenocarcinoma in distal lung epithelium has been developed in which transcription of TAg is driven by a lung specific 1011 base-pair DNA fragment of the rat Calbindin-D9K (CaBP9K) promoter [55]. In this model development of lung tumours was slower, with animals living to nearly a year, allowing analysis of early stages of tumour development.

In contrast to the TAg models which utilize lung promoter-viral oncogene fusion, human achaete-scute homolog-1 (hASH1) models have been developed that rely on the human transcription factor's fusion to the lung-specific clara cell 10 kDa secretory protein (CC10). Achaete-scute is a helix-loop-helix transcription factor involved in neural differentiation during fetal development. Neuroendocrine features are a hallmark of SCLC and some NSCLCs and the rationale behind development of this transgenic model was to investigate the effect of constitutive expression of achaete-scute in nonneuroendocrine airway epithelial cells that normally do not express it. Interestingly, expression resulted in the development of hyperplasia and bronchioloalveolar metaplasia. hASH1-CC10 was also generated in combination with the TAg oncogene to promote the growth of adenocarcinoma with neuroendocrine differentiation and increased tumourigenesis [52, 98]. These models typically resulted in tumour growth in 100 percent of animals, but exhibited rapid and aggressive growth which prevented the analysis of early transformation events.

Transgenic mice have also been generated through the fusion of oncogenes with lung-cell-specific promoters such as calcitonin gene-related peptide (CGRP), SP-C or CC10. CGRP-Ha-Ras transgenic mice overexpress an activated form of the GTPase, v-Ha-Ras, that induces pulmonary neuroendocrine cell differentiation [99]. The CGRP promoter limits transgene expression to neuroendocrine and neural cells. These transgenic mice surprisingly developed primary lung tumours which were non-neuroendocrine in nature along with hyperplasia of pulmonary neuroendocrine cells and Clara cells. This suggested a common histogenesis of different pulmonary cell types [56]. The Raf kinase protooncogene transduces signals downstream of Ras. It has been shown that mutations at the amino terminus of Raf that mediate its interactions with Ras can constitutively activate the Raf kinase activity such that it can transform cells in culture [100, 101]. Overexpression of wild-type Raf in tissue culture cells sensitizes the cells to Ras transformation [102] and analysis of human lung cancer cell lines and lung biopsy material have revealed increased levels of Raf expression, suggesting this might be related to development of lung cancer [103]. To investigate this in a transgenic model, mice were engineered to express c-Raf under the control of the SP-C promoter. Approximately half of the transgenic mice developed lung adenomas with delayed tumour development, suggesting that secondary mutations needed to be acquired before tumours could develop [104].

The protooncogene c-myc, normally involved in controlling cell-cycle events, has been frequently found to be over-expressed in human pulmonary carcinoids and adenocarcinomas [105, 106]. SP-C-Myc transgenic mice overexpress an activated form of the Myc protein that acts as a transcription factor, resulting in the development of bronchioloalveolar adenocarcinomas. Not all Myc transgenic mice develop lung cancer, again suggesting changes in addition to overexpression of Myc need to occur before cancer can develop [58]. SP-C-EML4-ALK transgenic mice possess EML4-ALK gene fusion specifically within the lung epithelial cells, resulting in rapid development of adenocarcinomas [57].

Genes encoding growth factors and growth factor receptors are also feasible targets for the generation of transgenic mice. SP-C-RON transgenic mice present with constitutive activation of the receptor tyrosine RON (recepteur d'origine nantais), localized by the SP-C promoter to distal lung epithelial cells, resulting in the development of adenoma and adenocarcinomas [60]. SP-C-IgEGF and SP-C-IgEGF-Myc transgenic lines express a secretable form of the epidermal growth factor (IgEGF), a structural and functional homologue of transforming growth factor *α* (TGF*α*). In the case of SP-C-IgEGF-Myc, additional expression of the murine oncogene, c-Myc, under the control of the SP-C promoter is initiated [58]. These transgenic lines develop alveolar hyperplasia and bronchioalveolar adenocarcinoma, respectively. Both the SP-C-RON and SP-C-IgEGF/Myc lines facilitate spatial expression of the transgene, but result in nonuniform metastasis and a characteristically poor response to therapy, confirming that these transgenic lines are ideal for examining the role of specific oncogenes in tumour growth, differentiation, and transformation but not in drug evaluation studies [58].

In order to streamline preexisting murine models and generate a more precise method of recapitulating true gene expression patterns of lung cancer oncogenes* in vivo*, conditional transgenic models have been created. Conditional transgenic models are ligand-inducible transgenic systems that result in regulated expression of the gene of interest through the use of two transgene constructs, one which acts as a target and one as the regulator. The regulator transgene must first be activated by the addition of an exogenous compound in order to turn on transcription of the target transgene [48, 49, 107, 108]. Conditional transgenic models allow for temporal and spatial regulation of oncogenes, providing a more accurate representation of the events that induce lung cancer.

There are three primary conditional bitransgenic inducible systems in mice. The first is the reverse tetracycline transactivator (rtTA) inducible system, in which a tissue specific promoter such as CCSP drives the expression of rtTA in the tissue of interest. A second transgene is incorporated containing the target gene, fused to the tetracycline-responsive promoter (Tet-O_7_). Expression of the target gene is then regulated by the addition of tetracycline or doxycycline [48, 51, 107]. Conditional bitransgenic rtTA systems used to study lung cancer are shown in Table 2. The majority of the rtTA models, including those expressing transgenes for K-Ras, EGFR, and FGF7, are valuable models in that a small number of cells can be targeted and transgene expression can be regulated both temporally and spatially. Interestingly, when doxycycline is removed from the K-Ras models, lesions can no longer be detected, indicating the importance of the K-Ras oncogene in both initial tumour growth and maintenance [108]. Despite these advantages, models expressing either K-Ras and FGF7 transgenes exhibit limited metastasis, failing to accurately mimic human adenocarcinoma* in vivo* [51, 108]. In contrast, models utilizing EGFR transgenes have been shown to metastasize early in development, resulting in early death of the animal and limited evaluation of early developmental events [48, 107].

An alternative to the rtTA system is the Cre/loxP recombination system, which facilitates the incorporation of somatic mutations in a select population of cells. The Cre/loxP system is ideal for examining both the conditional deletion of genes that cannot be examined in traditional knockout systems due to embryonic lethality, as well as the introduction of foreign genes in a tissue-specific manner. Cre is a 38 kDa recombinase protein that induces intramolecular and intermolecular recombination between loxP sites. A loxP site identifies the region for recombination, consisting of two 13 bp inverted repeats that are separated by an 8 bp asymmetric spacer region. Targeted mutations are “Floxed” (flagged by loxP sites) and through the addition of Cre recombinase, an endogenous gene or transgene is eliminated or activated by deletion of floxed sites [44–46, 50]. Current murine strains created using the Cre/loxP system are shown in Table 2. Cre transgenic strains can also be generated with Tet-inducible promoters [48]. The advantages of the Cre/loxP models are the ability to spatially regulate gene expression and evaluate events in lung cancer development [44–46].

Conditional transgenic strains resulting in conditional deletion of Trp53 alone and Trp53 in combination with pRb have proven to be one of the most valuable systems in modeling SCLC. Metastasis to select organs in these models has been shown to closely approximate metastatic events in humans as well as exhibit neuroendocrine features that are characteristic of human SCLC. Despite these advantages, these mice present with a very invasive phenotype, preventing examination of early transformation events [50].

Using the traditional Cre/loxP system, it is also possible to create a transcription block by floxing two sites in the region preceding an exon. The resulting null allele is dormant until Adeno-Cre is administered and the transcription stop is subsequently removed to allow for oncogenic mutation to occur. The lox-stop-lox (LSL) system is primarily used for K-RasG12D mutation in combination with other conditional knock-outs [47]. One of the most favored LSL models for lung cancer is the Lkb1:LSLK-RasG12D system which results in the development of adenocarcinoma and squamous cell carcinoma with metastasis that accurately reflects human metastatic events [47]. However, these strains often result in early death of the animal, and thus the system is not ideal for examining early transformation events.

## 6. Carcinogen-Inducible Models

In contrast to both transgenic and conditional transgenic systems, drug-induced models require the addition of a carcinogen to induce specific mutations leading to transformation events. The current carcinogen-inducible models for lung cancer are described in Table 2. Carcinogen-inducible models are typically generated in strains of inbred mice such as A/J or SWR which are most susceptible to spontaneous tumourigenesis [109]. Of these models, the urethane-induced lung tumourigenesis model has several advantages. Intraperitoneal administration of urethane has been shown to be reliably reproducible and subsequent tumourigenesis develops in a time-dependent manner. Tumourigenesis progresses from hyperplasia to adenoma and eventual adenocarcinoma in response to sequential genetic changes that are characteristic of human lung cancer [61]. Of these genetic changes, K-Ras and p53 are the most prominent mutations associated with the urethane-induced model [61, 110]. The benzo(a)pyrene-induced system also models adenoma in mice, however, it has been shown to result in extremely variable growth patterns in independent experiments [63]. N-Nitrosobis-(2-chloroethyl) ureas such as N-nitroso-methyl-bischloroethylurea (NMBCU) and N-nitroso-trischloroethylurea (NTCU) have been shown to induce the growth of hyperplasia, dysplasia and metaplasia following topical administration in Cr:NIH(S) mice [64]. 3-Methylcholanthrene, diethylnitrosamine, ethylnitrosourea, and dimethylhydrazine have all been shown to induce reproducible growth of adenoma in A/J mice [62]. Although these models provide the distinct advantage of investigator control of tumourigenesis through carcinogen administration, there are also multiple disadvantages associated with these models such as variability in administration technique leading to discrepancies in results.

## 7. Future Directions

To date, Xenograft models have been most commonly used to analyze the behavior of human tumours and their response to therapeutics in a mouse model. The use of genetically modified mice is perhaps a more powerful tool for studying lung cancer development and treatment but establishment of these models can be very laborious, expensive and time-consuming. A number of initiatives, both publicly and privately funded, have now been developed to create repositories of gene-targeting vectors, genetically modified mouse strains and predeveloped embryonic stem cells carrying specific mutations. Several of these sources are reviewed in Dow and Lowe [111]. The availability of these resources should significantly reduce the time required to generate new mouse models of lung cancer.

It is now evident that RNA interference can be used in mice to reduce or shut down specific gene expression, offering an alternative to traditional knockout models, which generally only affect one copy of a gene. Short hairpin RNAs (shRNAs) expressed transgenically act without integration into genomic material and operate in* trans* to affect expression of both copies of a gene. Depending on how the transgenic model is created, the silencing effects of shRNAs can be reversible, allowing disruption of gene expression in a temporal manner for investigation of effects at specific times during development. A fast and scalable method for developing shRNA transgenic mice has been recently used to validate p19^ARF^ as a therapeutic target for lung adenocarcinoma [112].

Embryonic stem cells (ESCs) have become another tool for rapid development of multiallelic mouse models. Multiple rounds of targeting disease-associated alleles in ESCs, followed by blastocysts injection and implantation, result in chimeric animals where tumours develop from the engineered cells in the context of a normal microenvironment. Chimeric animals may be cross-bred, generating wholly ESC derived mice. This methodology has been used to develop two different models of lung adenocarcinoma to analyze activation of pathways downstream of specific mutations and to assess the potential of therapeutic targeting strategies [112, 113].

A major criticism of using mouse models to model human cancer is inherent in the biological differences between the two organisms. In some cases, drugs that look promising for cancer therapy in a mouse model fail in clinical trials due to differences in activity between the mouse gene product being targeted and its human counterpart [114–116]. The effect of the human gene in transgenic mouse models can in some cases be most effectively addressed using humanized mice, in which a copy of the human gene replaces the mouse gene. Transgenic expression of the human cytochrome P450 2A13 was achieved using a cloned bacterial artificial chromosome in a background null for the mouse homologue. The results of this study indicated that the human gene was more highly effective at activating a carcinogenic compound present in cigarette smoke than its mouse homologue and in contributing to lung tumourigenesis [117]. There is a great deal of interest in finding useful predictive and prognostic serum or blood biomarkers for lung cancer patients as these fluids are easier and less painful to obtain than lung biopsies. Recently, Taguchi et al. found they could identify thirteen proteins overexpressed specifically in the plasma of mice bearing EGFR or Ras mutations that developed lung adenocarcinomas [118]. A subset of these proteins was measured in the serum of NSCLC patients and a significant concordance with the mouse data was found. Mice bearing the EGFR mutation and treated with the EGFR inhibitor erlotinib showed reduced levels of the markers associated with EGFR expression, similar to the human patients. This shows promise for the use of mouse models as a tool to identify new biomarkers.

## 8. Discussion

Taking into consideration each of the distinct preclinical models to study lung cancer* in vivo*, it is reasonable to conclude that each model is well-suited for a specific mode of study. For example, xenograft models are well-suited for the timely evaluation of response to therapy* in vivo*. However, transgenic and conditional transgenic model systems that accurately mimic tumour histology, genetic abnormalities and tumour microenvironment of human lung cancer, such as the LSL K-Ras G12D model for adenocarcinoma, and the Trp53 AdenoCre model for SCLC, may provide more reliable results concerning response to therapy and toxicity. To date, the majority of preclinical models used to evaluate efficacy of targeted chemotherapeutics are xenografts models, presumably due in part to the four to eight week growth period required to obtain results. Conversely, the use of xenograft models in preclinical study can lead to disappointing results in clinical trials. Current chemotherapies and the preclinical models used to evaluate them are summarized in Table 3.

Of the seventeen therapies summarized in Table 3, only three therapies were evaluated in transgenic or conditional transgenic murine models prior to progressing to clinical trial. In addition, only two of the therapies listed in Table 3 were assessed in a syngeneic, orthotopic model system. Both EGFR inhibitors, erlotinib and BIBW2992, were tested preclinically using the CCSP-rtTA; Tet-O_7_-EGFR^L858R^ model [26, 37, 49, 68, 70].* In vivo*, both therapies resulted in dramatic tumour regression, however, phase I clinical trials for BIBW2992 resulted in no significant partial or complete responses in patients [70]. On the other hand, clinical trials for erlotinib were successful in extending median survival to 8.4 months compared to a maximum of 8.0 months with gefitinib. One-year survival was increased to 40% compared to 37% with doxetaxel [68], suggesting that the model system was successful in predicting clinical benefit in the case of erlotinib, but not BIBW2992. Interestingly, the EGFR inhibitor vandetinib was evaluated* in vivo* using a H1975 xenograft model as opposed to the CCSP-rtTA; Tet-O_7_-EGFR^L858R^ model [119]. Vandetinib was found to significantly reduce tumour growth in the xenograft model, but resulted in limited response rates in clinical trials, which may be due in part to the fact that xenograft models cannot accurately recapitulate tumour microenvironment or predict immune response (Table 3). Thus, even the most complex murine models may predict clinical benefit of therapy in one case and not in another. Therefore, it is imperative that multiple models be used to evaluate efficacy of each therapy.

Syngeneic murine models prove to be reasonably successful in predicting clinical benefit of therapy in preclinical experiments (Table 3). The effects of navelbine and carboplatin were assessed in C57BL mice with LLC hind flank tumours.* In vivo*, IV navelbine administration resulted in 72.7 percent tumour regression [74]. Alternatively, IV carboplatin administration in combination with paclitaxel resulted in prolonged survival in 30–50 percent of the experimental population. Preclinical navelbine findings were shown to be translatable to clinical trials as median survival was extended to 34 weeks in patients [73]. Carboplatin-paclitaxel combination therapy was also shown to be effective in clinical trials as median survival was extended to 10.3 months in patients, further suggesting that the LLC model is a valuable tool for predicting clinical benefit of select therapies [81]. Interestingly, preclinical evaluation of monoclonal antibody therapy with bevacizumab and/or cetuximab has not been conducted in a syngeneic model system, but rather in xenograft systems [28, 85]. Both bevacizumab and cetuximab were shown to be effective in reducing tumour burden and extending survival both in preclinical and clinical trials [28, 84, 85, 88]. These studies raise several important questions concerning the translatability of preclinical study characteristics such as: clinically relevant dose, survival quantification and treatment regiment, to clinical trials.

It is important to note that preclinical and clinical dosages as well as treatment regimens vary widely between preclinical and clinical trials and even with the use of complex conditional transgenic models in preclinical studies, it is difficult to predict clinically relevant dose and appropriate treatment regimen for the patient population. There are several essential criteria for evaluating preclinical trial results prior to progression to clinical trials. Firstly, tumour growth inhibition of less than 50 percent in preclinical models does not typically translate into clinical benefit [69, 119]. Secondly, it is imperative that preclinical trial results show a survival benefit in response to therapy as this is one of the most telling criteria concerning drug efficacy* in vivo* [36, 79, 81]. Response to therapy cessation is also a valuable prognostic factor in preclinical studies. If therapy is discontinued and tumour growth resumes, relapse-free survival can be affected in patients and the likelihood of success in clinical trials may be limited [69, 119].

In summary, there are several valuable murine models available for the study of lung cancer; however, no one model can truthfully recapitulate all features of human lung cancer* in vivo*. Each model has both advantages and limitations and it is vital that these be taken into consideration prior to use in preclinical trials. Prior to choosing a model for experimentation, thought should be given to relevance of cell type, genetic abnormalities, temporal-spatial regulation of expression of target genes, tumour microenvironment, and the metastatic potential of each model. Despite recent advances, future research is needed, particularly with regards to developing models for SCLC and SCC as these are currently limited. Results obtained through the use of murine models as well as advancements in the development of new mouse models for lung cancer have provided much insight in the biology of lung cancer and lung cancer therapies. Ultimately, the use of these models in preclinical studies provides a vital framework from which to continue to evaluate therapies and identify predictive and prognostic markers* in vivo*.

## Figures and Tables

**Table 1:** Xenograft and orthotopic models for lung cancer.

Cell line	Description	Histology	Origin	Mutation	Adherence	Method of implantation	Animal model/s	Cell inoculation	Percentage tumor take	Palpable tumours	Metastatic potential	References
DMS-53	Small-cell carcinoma	SCLC	Male, age 54, smoking status unknown	Low Ras activity, p53 mutation	Adherent	Subcutaneous, hind flank injection	Female BALB/c nude mice Female nude athymic	10(6)–10(7) cells/mouse with PBS or matrigel	50–100%	4–8 weeks	Not previously described	[23, 24]

NCI-H69	Small-cell carcinoma	SCLC	Male, age 55, smoking status unknown	p53 deficient, wild type but low EGFR	Suspension, multicell aggregates	Subcutaneous, hind flank injection	Female athmyic nun/nu/CR hind flank	5 × 10(6) cells/mouse with PBS or matrigel	80–100%	15 days–4 weeks	Not previously described	[25]

A549	Adenocarcinoma	NSCLC	Male, age 58, smoking status unknown	Ras mutation	Adherent	Subcutaneous, hind flank injection	BALB /cAnNCrlBR athymic (*nu+/nu+*)	2 × 10^6^–2 × 10(7) cells/mouse with matrigel	100%	2–5 weeks	Not previously described	[26–30]
						Endobronchial	BALB/c or NMRI-nu/nu female mice	1 × 10(4)–1 × 10(6) cells/mouse	90%	9–61 days	Yes. Left lung, liver and spleen	[31]

H1299	Carcinoma	NSCLC	Male, age 43, smoking status unknown	N-Ras mutation, P53 negative High EGFR expression	Adherent	Subcutaneous, hind flank injection	Female athymic nude mice BALB/c nude mice	2 × 10^6^–1 × 10^7^ cells/mouse with matrigel	45–100%	4–6 weeks	Not previously described	[27, 29, 32]

NCI-H460	Large cell carcinoma	NSCLC	Male, age and smoking status unknown	K-Ras mutation	Adherent	Subcutaneous, hind flank injection	Female athymic nude mice (Ncr nu/nu)	3 × 10(5)–1 × 10(7) cells/mouse with matrigel	100%	4–11 days	Not previously described	[28, 31, 33–36]
						Endobronchial	Female athymic nude mice (Ncr nu/nu)	1 × 10(4)–1 × 10(6) cells/mouse	90%	9–61 days	Yes. Left lung, liver and spleen	[31]

H1975	Adenocarcinoma	NSCLC	Female, non-smoker	EGFR mutation L858R and T790M	Adherent	Subcutaneous, hind flank injection	Female athymic (nu/nu) mice NMRI-nu/nu female mice	1 × 10(6)–1 × 10(7) cells/mouse with matrigel	50–100%	4–8 weeks	Not previously described	[28, 37]

NCI-H226	Squamous carcinoma	NSCLC	Male, age and smoking status unknown	P53 mutation	Adherent	Subcutaneous, hind flank injection	BALB/c nude mice Female SCID/SCID mice	8 × 10^6^ cells/mouse with PBS or matrigel	50–100%	4–8 weeks	Not previously described	[36]

HCC827	Adenocarcinoma	NSCLC	Female, age 39, smoking status unknown	In-frame deletion (dE746-A750) in exon 19 of EGFR increased copy number	Adherent	Subcutaneous, hind flank injection	Female athymic (nu/nu) mice Female BALB/cA nude mice	2 × 10(7) cells/mouse with matrigel	80–100%	4-5 weeks	Not previously described	[26, 28]

HCC4006	Adenocarcinoma	NSCLC	Male, age >50, smoking status unknown	In-frame deletion (dE746-A750) in exon 19 of EGFR	Adherent	Subcutaneous, hind flank injection	Female SCID/SCID mice	1-2 × 10^6^ cells/mouse with PBS or matrigel	50–100%	4–8 weeks	Not previously described	[38]

NCI-H358	Bronchioalviolar carcinoma	NSCLC	Male, age and smoking status unknown	Wild-type EGFR	Adherent	Subcutaneous, hind flank injection	Female athymic (nu/nu) mice	2 × 10^7^ cells/mouse with PBS or matrigel	100%	4-5 weeks	Not previously described	[26, 28, 39]

LLC	Lewis lung carcinoma	NSCLC	C57BL mouse	Not reported	Mixed, adherent and suspension	Subcutaneous, leg injection	Male C57BL mice	2 × 10^6^ cells/mouse	100%	7 days	Yes. Lung	[40]

NCI-H23	Adenocarcinoma	NSCLC	Male, 51 years	p53 mutation	Adherent	Subcutaneous	Female BALB-C nude mice	3 × 3 × 3 mm tumor fragment	100%	14 days	Not previously described	[36]

DMS-273	Small-cell carcinoma	SCLC	Female, 50 years	p53 mutation	Adherent	Subcutaneous	Female BALB-C nude mice	3 × 3 × 3 mm tumor fragment	100%	14 days	Not previously described	[36]

DMS-114	Small-cell carcinoma	SCLC	Male, 68 years	p53 mutation	Adherent	Subcutaneous	Female BALB-C nude mice	3 × 3 × 3 mm tumor fragment	100%	14 days	Not previously described	[36]

TL-1	Squamous carcinoma	NSCLC	Not reported	Not reported	Adherent	Subcutaneous	CB-17 scid/scid mice	2 × 10^6^ cells/mouse in saline	60–70%	3-4 weeks	Not previously described	[41]

NCI-H526	Carcinoma	SCLC	Male, 55 years	KIT positive	Suspension	Subcutaneous, hind flank injection	Female athymic nu/nu mice	5 × 10^6^ cells/mouse in saline	100%	20 days	Not previously described	[42]

NCI H82	Carcinoma	SCLC	Male, 40 years	KIT negative	Suspension	Subcutaneous, hind flank injection	Female athymic nu/nu mice	5 × 10^6^ cells/mouse in saline	100%	25 days	Not previously described	[42]

NCI-H358	Bronchioalviolar carcinoma	NSCLC	Male	Wt EGFR Mutated ras	Adherent	Subcutaneous, hind flank injection	Female athymic nu/nu mice	2 × 10^7^ cells/mouse with 1 : 1 PBS and matrigel	100%	Not reported	Not previously described	[28]

**Table 2:** Transgenic, conditional transgenic and carcinogen-inducible models for lung cancer (^∗^intratracheal instillation of Ad-Cre virus is required; TAg: simian virus T antigen; CCSP: clara cell secretory protein; SP-C: alveolar type II surfactant protein C; CaBP9K: rat calbindin-D9K; CC10: clara cell 10 kDa secretory protein; CGRP: calcitonin gene-related peptide; rtTA-reverse tetracycline transactivator protein; LSL: lox-stop-lox; hASH1: human achaete-scute homolog 1; EGFR: epidermal growth factor receptor; FGF: fibroblast growth factor).

Model type	Model	Background	Histology	Advantages	Disadvantages	References
*Conditional *						
Oncogenes	LSL K-Ras G12D^*^ K-Ras4b G12D^*^ K-Ras V12^*^	C57BL/6 C57BL/6 C57BL/6	Adenocarcinoma Adenocarcinoma Adenocarcinoma	(i) Limited number of cells can be targeted (ii) Ideal for studying early lung tumor development	(i) Limited metastasis (ii) (One mutation not sufficient to produce higher grade malignancy) (iii) Gene expression signature slightly different for human K-Ras	[44] [45] [46]
	Lkb1: LSL K-Ras^G12D^	C57BL/6	Adenocarcinoma/squamous cell carcinoma, large cell carcinoma in some cases	(i) Metastasis (ii) Limited number of cells can be targeted	(i) Very invasive, often early death of animal	[47]

Growth factor receptors	CCSP-rtTA; Tet-0_7_-EGFR^L858R^ CCSP-rtTA; Tet-0_7_-EGFR^DEL^	FVB/N FVB/N	Bronchioalveolar carcinoma Bronchioalveolar carcinoma	(i) Turning on and off with tetracycline/doxycycline (ii) Can target expression to pulmonary epithelium (iii) Resemblance to human adenocarcinoma	(ii) Invasive metastasis within 4 weeks (iii) More difficult to study early cancer events	[48, 49]

Tumor suppressor genes	Trp53^*^ Rb-Trp53^*^	C57/BL/6 or 129/Sv wild-type Athymic BALB/c nu/nu	Adenocarcinoma SCLC Neuroendocrine hyperplasia, SCLC	(i) Turning expression on and off (ii) Metastasis towards similar organs as human SCLC (iii) Share neuroendocrine features of SCLC	(i) Very invasive, often early death of animal (ii) More difficult to study early cancer events	[50]

Growth factors	CC10-rtTA; Tet-0_7_-CMV-FGF7	CBA/C57Bl6	Epithelial cell hyperplasia and adenomatous hyperplasia	(i) Hyperplasia disappears when doxycycline removed	(i) Limited metastasis (fails to accurately mimic human adenocarcinoma)	[51]

*Transgenic *						
Transcription factors	CC10-hASH1 CC10-Tag; CC10-hASH1	FVB FVB	Hyperplasia and bronchioloaleveolar metaplasia Adenocarcinoma with neuroendocrine differentiation	(i) Ideal for carcinogenesis and cancer prevention studies and the role of specific oncogene in growth, differentiation, transformation	(i) Measurable in late stage (ii) Metastasis not uniform (iii) Response to therapy is typically poor	[52]

Viral oncogenes	SP-C-TAg CCSP-TAg CaBP9K-TAg	FVB/N FVB/N FVB/N	Adenocarcinoma Adenocarcinoma Adenocarcinoma	(i) Typically 100% tumor take (ii) Ideal for carcinogenesis and cancer prevention studies	(i) Rapid onset and aggressive (ii) Difficult to examine early events in transformation (iii) Difficult to detect events independent of oncogene expression	[53] [54] [55]

Oncogenes	CGRP-H-Ras	FVB/N	Neuroendocrine hyperplasia and non-neuorendocrine adenocarcinoma	(i) Ideal for carcinogenesis and cancer prevention studies and the role of specific oncogene in growth, differentiation, and transformation	(i) Often early death of animal (ii) Relationship between Ras isoforms roles in transformation not fully understood	[56]
	SP-C-EML4-ALK	C57BL/6J	Adenocarcinoma	(i) Aggressive presentation (ii) Ideal for carcinogenesis and cancer prevention studies	(i) Often early death of animal (ii) No conditional expression	[57]
	SP-C-Myc CC10-Myc	CD2/F1 (DBA/2 × Balb/C) CD2/F1 (DBA/2 × Balb/C)	Adenocarcinoma Bronchioloalveolar hyperplasia	(i) Aggressive presentation (ii) Ideal for carcinogenesis and cancer prevention studies	(i) Do not often metastasize (ii) Metastasis not uniform (iii) Response to therapy is typically poor	[58]
	SP-C-cRaf-1 SP-C-c-Raf-1-BxB	C57BL/6 × DBA-2 C57BL/6 × DBA-2	Adenoma Adenoma	(i) Aggressive presentation (ii) Ideal for carcinogenesis and cancer prevention studies	(i) Do not often metastasize (ii) Measurable in late stage (iii) Metastasis not uniform (iv) Response to therapy is typically poor	[59]

Growth factors	SP-C-RON	B6C3/F1 hybrid mice (C57BL/6 × C3H, Taconic)	Adenoma and adenocarcinoma	(i) Temporal-spatial expression (ii) Ideal for carcinogenesis and cancer prevention studies	(i) Metastasis not uniform (ii) Response to therapy is typically poor	[60]

Growth factor receptors	SP-C-IgEGF SP-C-cMyc; SpC-IgEGF)	CD2/F1 (DBA/2 × Balb/C) CD2/F1 (DBA/2 × Balb/C)	Alveolar hyperplasia Bronchioloalvealor Adenocarcinoma	(i) Ideal for carcinogenesis and cancer prevention studies	(i) Metastasis not uniform (ii) Response to therapy is typically poor	[58]

Carcinogen inducible models	Inoculation Method	Background	Growth Properties	Advantages	Disadvantages	References

*Urethane*	Intraperitoneal	Inbred (A/J or SWR most widely used)	Adenoma	(i) More likely to accurately predict clinical efficacy of chemotherapeutic agents (ii) Aggressive (iii) All tumor stages can be observed	(i) Lethality (ii) Low metastatic potential (iii) Varying response to carcinogen (iv) Low rate of spontaneous development (v) Long incubation time	[61]
Benzo(a)pyrene	Intraperitoneal		Adenoma			[62, 63]
N-Nitrosobis-(2-cloroethyl) ureas	Topical	Cr:NIH(S)	Adenosquamous carcinoma			[64]
Dimethylhydrazine	Intraperitoneal	A/J	Adenoma			[62]
DiethylInitrosamine	Intraperitoneal		Adenoma			
EthylInitrosourea	Intraperitoneal		Adenoma			
3-Methylchholanthrene	Intraperitoneal		Adenoma			

**Table 3:** Preclinical and clinical trial summaries for lung cancer therapies (CCSP: clara cell secretory protein; rtTA: reverse tetracycline transactivator protein; EGFR: epidermal growth factor receptor; RR: response rate; PFS: progression free survival).

Drug name	Route	Mechanism	Preclinical dosage	Preclinical frequency	FDA approval	Preclinical model	Preclinical results	Clinical Dosage	Clinical Frequency	Results of Clinical Trials	References
Gefitinib	Oral	HER1/EGFR inhibitor	0.01 *μ*M 50–200 mg/kg	qd × 5 days × 2 weeks	2009	**Xenograft**-A549 cells in athymic nude	Tumor regression and increase in median survival	250 mg	qd	Positive	[66, 67]

Erlotinib	Oral	HER1/EGFR inhibition	0.5% (w/v) I.P. or 25 mg/kg/day	qd × 5 days × q4 weeks	2005	**Xenograft**-HCC827, A549, NCI358 cells in female BALB/cA nude mice *Transgenic*-CCSP-rtTA; Tet-0_7_-EGFR^L858R^	Tumor regression observed in HCC827 xenografts	150 mg	qd	Positive	[26, 49, 68]

Vandetanib	Oral	VEGF/EGFR inhibitor	25 mg/kg	qd	Not approved	**Xenograft-**H1975 cells in female athymic nude mice	Inhibition of tumor growth (not dramatic)	50–145 mg/m^2^	qd	Negative	[69]

BIBW2992	Oral	HER2/EGFR inhibitor	20 mg/kg	qd	October 2010 Phase III clinical trial	**Xenograft-**H1975 cells in female athmyic NMRI-nu/nu mice **Transgenic**-CCSP-rtTA; Tet-0_7_-EGFR^L858R^	Dramatic tumor regression T/C ratio 2%	20–70 mg	D1, 8, 15 q4 weeks	Positive	[37, 70]

Crizotinib	Oral	ALK inhibitor	10 mg/kg	qd	Phase III clinical trials	**Transgenic-**SP-C-EML4-ALK in C57BL/6J mice	Tumor regression and increase in median survival	250 mg	2 d × 6 months	Positive	[57, 71, 72]

Navelbine (Vinorelbine)	Oral, IV	Antimicrotubule chemotherapy	1.25–5 mg/kg	qd × 9 days	1994	**Immune-**C57B1 mice used for transplantation of LLC	72.7% inhibition of tumor growth	25–30 mg/m^2^	q weekly	Positive	[73, 74]

Paclitaxel	IV	Antimicrotubule chemotherapy	12–24 mg/kg	qd × 5 days	1992	**Xenograft**-A549, NCI-H23, NCI-H460, DMS-273, NCI-H226, and DMS-114 cells in nude mice	Significant tumor regression, more effective than cisplatin	135 mg/m^2^	q3 weeks	Positive	[36, 75]

Abraxane	IV	Antimicrotubule chemotherapy	250 mg/kg IP	qd × 3 weeks	Phase III clinical trials for lung cancer	**Xenograft-**H460 cells in female athymic nu/nu mice	Significant tumor regression	260 mg/m^2^	q3 weeks	Positive	[23, 34]

Gemcitabine	IV	Nucleoside analog chemotherapy	50–160 mg/kg	q3-4 days	2006	**Xenograft**-A549 and H1299 cells in female nude mice	Reduced mean tumor double time by 50% in 13 days	1000 mg/m^2^	D1, 8, 15 q28 days	Positive	[32, 76]

Pemetrexed	IV	Folate antimetabolite chemotherapy	30 mg/kg	q3-4 days	2004	**Xenograft-**H460 cells in female athymic nude	Tumor regression duration and dose dependent	500 mg/m^2^	q21 days	Positive	[35, 77]

Doxorubicin	IV	Anthracycline antibiotic chemotherapy	3.0–12 mg/kg	qd	1950's	**Xenograft-**Nu/nu-Balb/c/ABom, normal Balb/c (13) CB-17 scid/scid with TL-1 s.c. 2 × 10(6) cells in 0.2mL saline (14)	Effective in arresting tumor growth	40–75 g/m^2^	q21–28 days	Positive	[41, 78]

Cisplatin	IV	Platinum-based chemotherapy	7 mg/kg I.P	qd × 2 weeks q4 weeks	1969	**Xenograft**-A549, H1299 nude mice **Transgenic**-LSLK-RasG12D on 129svJae background	Significantly reduced tumor burden, but left long-term resistance	60 to 100 mg/m^2^	q21 days	Positive	[32, 79, 80]

Carboplatin	IV	Platinum-based chemotherapy	50 mg/kg I.P.	qd	1989	**Xenograft-**A549 and H1299 cells in athmyic nude mice **Immune-**C57B1 mice used for transplantation of LLC	Two-drug regimen response rates 30–50% prolonged median survival of >1 year	200–360 mg/m^2^	q4 weeks	Positive	[32, 40, 81]

Etoposide	IV, oral	DNA topoisomerase II inhibitor	1–32 mg/kg	qd × 5 days	1960's	**Xenograft-**H460 cells in female athmyic nude mice	Tumor growth regression	100 mg/m^2^ IV 200 mg/m^2^ PO	q3 weeks	Positive	[82, 83]

Bevacizumab	IV	Monoclonal antibody VEGF-A inhibitor	5 mg/kg	qd × 4 weeks	2004	**Xenograft-**H1299 cells in athymic BALB/c female nude mice	Reduced vascularity, reduced interstitial pressure and tumor growth	15 mg/kg	q3 weeks	Positive	[84, 85]

Sunitinib	oral	Multitargeted RTK inhibitor (VEGF, CKit, PDGF*α*, cRET)	40 mg/kg	qd × 6 weeks	Phase II clinical trials for lung cancer	**Xenograft-**NCI-H226, NCI-H526 or NCI-H82 in athmyic female nu/nu mice	Significant tumor growth regression	37.5–50 mg	continuous qd or qd × 4 weeks q6 weeks	Negative	[42, 86]

Sorafinib	oral	Multi-targeted RTK inhibitor (VEGF, CKit, PDGF*α*, cRET)	40–80 mg/kg	qd × 9 days	Phase III clinical trials for lung cancer	**Xenograft-**H460, A549, NCI-H23 cells in female NCr-nu/nu mice	Significant tumor growth regression	400 mg	bid	Negative	[33, 87]

Cetuximab	IV	Monoclonal antibody EGFR inhibitor	20 *μ*L/g	q3 weeks	2008	**Xenograft-**A549, NCI-H358, HCC-827, H1975, H460 cells in female athmyic nu/nu mice	Significant tumor growth inhibition	250–400 mg/m^2^	400 mg/m^2^— 250 mg/m^2^ q weekly	Negative	[28, 88]

## References

[1] Molina J. R., Yang P., Cassivi S. D., Schild S. E., Adjei A. A. (2008). Non-small cell lung cancer: epidemiology, risk factors, treatment, and survivorship. *Mayo Clinic Proceedings*.

[2] Siegel R., Naishadham D., Jemal A. (2013). Cancer statistics, 2013. *CA: A Cancer Journal for Clinicians*.

[3] Mao L., Lee J. S., Kurie J. M. (1997). Clonal genetic alterations in the lungs of current and former smokers. *Journal of the National Cancer Institute*.

[4] Todd S., Franklin W. A., Varella-Garcia M. (1997). Homozygous deletions of human chromosome 3p in lung tumors. *Cancer Research*.

[5] Belinsky S. A., Klinge D. M., Stidley C. A. (2003). Inhibition of DNA methylation and histone deacetylation prevents murine lung cancer. *Cancer Research*.

[6] Dacic S., Flanagan M., Cieply K. (2006). Significance of EGFR protein expression and gene amplification in non-small cell lung carcinoma. *American Journal of Clinical Pathology*.

[7] Herbst R. S., Heymach J. V., Lippman S. M. (2008). Molecular origins of cancer: lung cancer. *The New England Journal of Medicine*.

[8] Marks J. L., Broderick S., Zhou Q. (2008). Prognostic and therapeutic implications of EGFR and KRAS mutations in resected lung adenocarcinoma. *Journal of Thoracic Oncology*.

[9] Perner S., Wagner P. L., Demichelis F. (2008). EML4-ALK fusion lung cancer: a rare acquired event. *Neoplasia*.

[10] Pao W., Miller V., Zakowski M. (2004). EGF receptor gene mutations are common in lung cancers from “never smokers” and are associated with sensitivity of tumors to gefitinib and erlotinib. *Proceedings of the National Academy of Sciences of the United States of America*.

[11] Rosell R., Moran T., Queralt C. (2009). Screening for epidermal growth factor receptor mutations in lung cancer. *The New England Journal of Medicine*.

[12] Riely G. J., Kris M. G., Rosenbaum D. (2008). Frequency and distinctive spectrum of KRAS mutations in never smokers with lung adenocarcinoma. *Clinical Cancer Research*.

[13] Pao W., Girard N. (2011). New driver mutations in non-small-cell lung cancer. *The Lancet Oncology*.

[14] Massarelli E., Varella-Garcia M., Tang X. (2007). *KRAS* mutation is an important predictor of resistance to therapy with epidermal growth factor receptor tyrosine kinase inhibitors in non-small cell lung cancer. *Clinical Cancer Research*.

[15] Fukasawa K., Choi T., Kuriyama R., Rulong S., Woude G. F. V. (1996). Abnormal centrosome amplification in the absence of p53. *Science*.

[16] Rodin S. N., Rodin A. S. (2000). Human lung cancer and p53: the interplay between mutagenesis and selection. *Proceedings of the National Academy of Sciences of the United States of America*.

[17] Steels E., Paesmans M., Berghmans T. (2001). Role of p53 as a prognostic factor for survival in lung cancer: a systematic review of the literature with a meta-analysis. *European Respiratory Journal*.

[18] Sève P., Mackey J., Isaac S. (2005). Class III *β*-tubulin expression in tumor cells predicts response and outcome in patients with non-small cell lung cancer receiving paclitaxel. *Molecular Cancer Therapeutics*.

[19] Zheng Z., Chen T., Li X., Haura E., Sharma A., Bepler G. (2007). DNA synthesis and repair genes RRM1 and ERCC1 in lung cancer. *The New England Journal of Medicine*.

[20] Kwei K. A., Kim Y. H., Girard L. (2008). Genomic profiling identifies TITF1 as a lineage-specific oncogene amplified in lung cancer. *Oncogene*.

[21] Gadgeel S. M., Wozniak A. (2013). Preclinical rationale for PI3K/Akt/mTOR pathway inhibitors as therapy for epidermal growth factor receptor inhibitor-resistant non-small-cell lung cancer. *Clinical Lung Cancer*.

[22] Han D., Li S.-J., Zhu Y.-T., Liu L., Li M.-X. (2013). LKB1/AMPK/mTOR signaling pathway in non-small-cell lung cancer. *Asian Pacific Journal of Cancer Prevention*.

[23] Memon A. A., Jakobsen S., Dagnaes-Hansen F., Sorensen B. S., Keiding S., Nexo E. (2009). Positron emission tomography (PET) imaging with [^11^C]-labeled erlotinib: a micro-PET study on mice with lung tumor xenografts. *Cancer Research*.

[24] Steiner P., Joynes C., Bassi R. (2007). Tumor growth inhibition with cetuximab and chemotherapy in non-small cell lung cancer xenografts expressing wild-type and mutated epidermal growth factor receptor. *Clinical Cancer Research*.

[25] Sakuma Y., Matsukuma S., Nakamura Y. (2013). Enhanced autophagy is required for survival in EGFR-independent EGFR-mutant lung adenocarcinoma cells. *Laboratory Investigation*.

[26] Su H., Bodenstein C., Dumont R. A. (2006). Monitoring tumor glucose utilization by positron emission tomography for the prediction of treatment response to epidermal growth factor receptor kinase inhibitors. *Clinical Cancer Research*.

[27] Li D., Ambrogio L., Shimamura T. (2008). BIBW2992, an irreversible EGFR/HER2 inhibitor highly effective in preclinical lung cancer models. *Oncogene*.

[28] Akhtar S., Meeran S. M., Katiyar N., Katiyar S. K. (2009). Grape seed proanthocyanidins inhibit the growth of human non-small cell lung cancer xenografts by targeting insulin-like growth factor binding protein-3, tumor cell proliferation, and angiogenic factors. *Clinical Cancer Research*.

[29] Chen M.-F., Chen W.-C., Wu C.-T. (2006). p53 status is a major determinant of effects of decreasing peroxiredoxin I expression on tumor growth and response of lung cancer cells to treatment. *International Journal of Radiation Oncology, Biology, Physics*.

[30] Wang H., Li M., Rinehart J. J., Zhang R. (2004). Pretreatment with dexamethasone increases antitumor activity of carboplatin and gemcitabine in mice bearing human cancer xenografts: *in vivo* activity, pharmacokinetics, and clinical implications for cancer chemotherapy. *Clinical Cancer Research*.

[31] Carter C. A., Chen C., Brink C. (2007). Sorafenib is efficacious and tolerated in combination with cytotoxic or cytostatic agents in preclinical models of human non-small cell lung carcinoma. *Cancer Chemotherapy and Pharmacology*.

[32] Feng Z., Zhao G., Yu L., Gough D., Howell S. B. (2010). Preclinical efficacy studies of a novel nanoparticle-based formulation of paclitaxel that out-performs Abraxane. *Cancer Chemotherapy and Pharmacology*.

[33] McLemore T. L., Liu M. C., Blacker P. C. (1987). Novel intrapulmonary model for orthotopic propagation of human lung cancers in athymic nude mice. *Cancer Research*.

[34] Teicher B. A., Chen V., Shih C. (2000). Treatment regimens including the multitargeted antifolate LY231514 in human tumor xenografts. *Clinical Cancer Research*.

[35] Yamori T., Sato S., Chikazawa H., Kadota T. (1997). Anti-tumor efficacy of paclitaxel against human lung cancer xenografts. *Japanese Journal of Cancer Research*.

[36] Qin M., Chen S., Yu T., Escuadro B., Sharma S., Batra R. K. (2003). Coxsackievirus adenovirus receptor expression predicts the efficiency of adenoviral gene transfer into non-small cell lung cancer xenografts. *Clinical Cancer Research*.

[37] Pettengill O. S., Sorenson G. D., Wurster-Hill D. H. (1980). Isolation and growth characteristics of continuous cell lines from small-cell carcinoma of the lung. *Cancer*.

[38] Taylor J. E., Bogden A. E., Moreau J.-P., Coy D. H. (1988). *In vitro* and *in vivo* inhibition of human small cell lung carcinoma (NCI-H69) growth by a somatostatin analogue. *Biochemical and Biophysical Research Communications*.

[39] Rygaard K., Vindelov L. L., Spang-Thomsen M. (1993). Expression of myc family oncoproteins in small-cell lung-cancer cell lines and xenografts. *International Journal of Cancer*.

[40] Fichtner I., Rolff J., Soong R. (2008). Establishment of patient-derived non-small cell lung cancer xenografts as models for the identification of predictive biomarkers. *Clinical Cancer Research*.

[41] Merk J., Rolff J., Becker M., Leschber G., Fichtner I. (2009). Patient-derived xenografts of non-small-cell lung cancer: a pre-clinical model to evaluate adjuvant chemotherapy?. *European Journal of Cardio-thoracic Surgery*.

[42] Dong X., Guan J., English J. C. (2010). Patient-derived first generation xenografts of non-small cell lung cancers: promising tools for predicting drug responses for personalized chemotherapy. *Clinical Cancer Research*.

[43] Nogawa M., Yuasa T., Kimura S. (2005). Monitoring luciferase-labeled cancer cell growth and metastasis in different in vivo models. *Cancer Letters*.

[44] John T., Kohler D., Pintilie M. (2011). The ability to form primary tumor xenografts is predictive of increased risk of disease recurrence in early-stage non-small cell lung cancer. *Clinical Cancer Research*.

[45] Johnson D. H., Paul D. M., Hande K. R., DeVore R. (1996). Paclitaxel plus carboplatin in the treatment of patients with advanced lung cancer: a Vanderbilt University Cancer Center phase II trial (LUN-46). *Seminars in Oncology*.

[46] Van Moorsel C. J. A., Pinedo H. M., Veerman G., Vermorken J. B., Postmus P. E., Peters G. J. (1999). Scheduling of gemcitabine and cisplatin in Lewis Lung tumour bearing mice. *European Journal of Cancer*.

[47] Sandler A., Gray R., Perry M. C. (2006). Paclitaxel-carboplatin alone or with bevacizumab for non-small-cell lung cancer. *The New England Journal of Medicine*.

[48] Sakai Y., Sasahira T., Ohmori H., Yoshida K., Kuniyasu H. (2006). Conjugated linoleic acid reduced metastasized LL2 tumors in mouse peritoneum. *Virchows Archiv*.

[49] Papageorgiou A., Stravoravdi P., Sahpazidou D., Natsis K., Chrysogelou E., Toliou T. (2000). Effect of Navelbine on inhibition of tumor growth, cellular differentiation and estrogen receptor status on Lewis lung carcinoma. *Chemotherapy*.

[50] Marty M., Fumoleau P., Adenis A. (2001). Oral vinorelbine pharmacokinetics and absolute bioavailability study in patients with solid tumors. *Annals of Oncology*.

[51] Herbst R. S., Takeuchi H., Teicher B. A. (1998). Paclitaxel/carboplatin administration along with antiangiogenic therapy in non-small-cell lung and breast carcinoma models. *Cancer Chemotherapy and Pharmacology*.

[52] Zhao B., Magdaleno S., Chua S. (2000). Transgenic mouse models for lung cancer. *Experimental Lung Research*.

[53] Virmani A. K., Gazdar A. F. (2003). Tumor suppressor genes in lung cancer. *Methods in Molecular Biology*.

[54] DeMayo F. J., Finegold M. J., Hansen T. N., Stanley L. A., Smith B., Bullock D. W. (1991). Expression of SV40 T antigen under control of rabbit uteroglobin promoter in transgenic mice. *American Journal of Physiology—Lung Cellular and Molecular Physiology*.

[55] Wikenheiser K. A., Clark J. C., Linnoila R. I., Stahlman M. T., Whitsett J. A. (1992). Simian virus 40 large T antigen directed by transcriptional elements of the human surfactant protein C gene produces pulmonary adenocarcinomas in transgenic mice. *Cancer Research*.

[56] Chailley-Heu B., Rambaud C., Barlier-Mur A.-M. (2001). A model of pulmonary adenocarcinoma in transgenic mice expressing the simian virus 40 T antigen driven by the rat Calbindin-D9K (CaBP9K) promoter. *The Journal of Pathology*.

[57] Linnoila R. I., Sahu A., Miki M., Ball D. W., DeMayo F. J. (2000). Morphometric analysis of CC10-hASH1 transgenic mouse lung: a model for bronchiolization of alveoli and neuroendocrine carcinoma. *Experimental Lung Research*.

[58] Linnoila R. I., Zhao B., DeMayo J. L. (2000). Constitutive achaete-scute homologue-1 promotes airway dysplasia and lung neuroendocrine tumors in transgenic mice. *Cancer Research*.

[59] Mabry M., Nakagawa T., Baylin S., Pettengill O., Sorenson G., Nelkin B. (1989). Insertion of the v-Ha-ras oncogene induces differentiation of calcitonin-producing human small cell lung cancer. *The Journal of Clinical Investigation*.

[60] Sunday M. E., Haley K. J., Sikorski K. (1999). Calcitonin driven v-Ha-ras induces multilineage pulmonary epithelial hyperplasias and neoplasms. *Oncogene*.

[61] Stanton V. P., Nichols D. W., Laudano A. P., Cooper G. M. (1989). Definition of the human raf amino-terminal regulatory region by deletion mutagenesis. *Molecular and Cellular Biology*.

[62] Heidecker G., Huleihel M., Cleveland J. L. (1990). Mutational activation of c-raf-1 and definition of the minimal transforming sequence. *Molecular and Cellular Biology*.

[63] Cuadrado A., Bruder J. T., Heidaran M. A., App H., Rapp U. R., Aaronson S. A. (1993). H-ras and raf-1 cooperate in transformation of NIH3T3 fibroblasts. *Oncogene*.

[64] Rapp U. R., Huleihel M., Pawson T. (1988). Role of raf oncogenes in lung carcinogenesis. *Lung Cancer*.

[65] Kerkhoff E., Fedorov L. M., Siefken R., Walter A. O., Papadopoulos T., Rapp U. R. (2000). Lung-targeted expression of the c-Raf-1 kinase in transgenic mice exposes a novel oncogenic character of the wild-type protein. *Cell Growth and Differentiation*.

[66] Broers J. L., Viallet J., Jensen S. M. (1993). Expression of c-myc in progenitor cells of the bronchopulmonary epithelium and in a large number of non-small cell lung cancers. *The American Journal of Respiratory Cell and Molecular Biology*.

[67] Lorenz J., Friedberg T., Paulus R., Oesch F., Ferlinz R. (1994). Oncogene overexpression in non-small-cell lung cancer tissue: prevalence and clinicopathological significance. *Clinical Investigator*.

[68] Ehrhardt A., Bartels T., Geick A., Klocke R., Paul D., Halter R. (2001). Development of pulmonary bronchiolo-alveolar adenocarcinomas in transgenic mice overexpressing murine c-myc and epidermal growth factor in alveolar type II pneumocytes. *British Journal of Cancer*.

[69] Soda M., Takada S., Takeuchi K. (2008). A mouse model for EML4-ALK-positive lung cancer. *Proceedings of the National Academy of Sciences of the United States of America*.

[70] Chen Y.-Q., Zhou Y.-Q., Fu L.-H., Wang D., Wang M.-H. (2002). Multiple pulmonary adenomas in the lung of transgenic mice overexpressing the RON receptor tyrosine kinase. *Carcinogenesis*.

[71] Perl A.-K. T., Tichelaar J. W., Whitsett J. A. (2002). Conditional gene expression in the respiratory epithelium of the mouse. *Transgenic Research*.

[72] Politi K., Fan P.-D., Shen R., Zakowski M., Varmus H. (2010). Erlotinib resistance in mouse models of epidermal growth factor receptor-induced lung adenocarcinoma. *Disease Models & Mechanisms*.

[73] Politi K., Zakowski M. F., Fan P.-D., Schonfeld E. A., Pao W., Varmus H. E. (2006). Lung adenocarcinomas induced in mice by mutant EGF receptors found in human lung cancers respond to a tyrosine kinase inhibitor or to down-regulation of the receptors. *Genes and Development*.

[74] Fisher G. H., Wellen S. L., Klimstra D. (2001). Induction and apoptotic regression of lung adenocarcinomas by regulation of a K-Ras transgene in the presence and absence of tumor suppressor genes. *Genes and Development*.

[75] Tichelaar J. W., Lu W., Whitsett J. A. (2000). Conditional expression of fibroblast growth factor-7 in the developing and mature lung. *The Journal of Biological Chemistry*.

[76] Meuwissen R., Linn S. C., van der Valk M., Mooi W. J., Berns A. (2001). Mouse model for lung tumorigenesis through Cre/lox controlled sporadic activation of the K-Ras oncogene. *Oncogene*.

[77] Meuwissen R., Linn S. C., Linnoila R. I., Zevenhoven J., Mooi W. J., Berns A. (2003). Induction of small cell lung cancer by somatic inactivation of both Trp53 and Rb1 in a conditional mouse model. *Cancer Cell*.

[78] Jackson E. L., Willis N., Mercer K. (2001). Analysis of lung tumor initiation and progression using conditional expression of oncogenic K-ras. *Genes and Development*.

[79] Tuveson D. A., Shaw A. T., Willis N. A. (2004). Endogenous oncogenic K-ras(G12D) stimulates proliferation and widespread neoplastic and developmental defects. *Cancer Cell*.

[80] Ji H., Ramsey M. R., Hayes D. N. (2007). LKB1 modulates lung cancer differentiation and metastasis. *Nature*.

[81] de Seranno S., Meuwissen R. (2010). Progress and applications of mouse models for human lung cancer. *European Respiratory Journal*.

[82] Ohno J., Horio Y., Sekido Y. (2001). Telomerase activation and p53 mutations in urethane-induced A/J mouse lung tumor development. *Carcinogenesis*.

[83] Horio Y., Chen A., Rice P., Roth J. A., Malkinson A. M., Schrump D. S. (1996). Ki-ras and p53 mutations are early and late events, respectively, in urethane-induced pulmonary carcinogenesis in A/J mice. *Molecular Carcinogenesis*.

[84] Gunning W. T., Kramer P. M., Lubet R. A. (2003). Chemoprevention of benzo(a)pyrene-induced lung tumors in mice by the farnesyltransferase inhibitor R115777. *Clinical Cancer Research*.

[85] Rehm S., Lijinsky W., Singh G., Katyal S. L. (1991). Mouse bronchiolar cell carcinogenesis. Histologic characterization and expression of Clara cell antigen in lesions induced by N-nitrosobis-(2-chloroethyl) ureas. *The American Journal of Pathology*.

[86] Stoner G. D., Greisiger E. A., Schut H. A. J. (1984). A comparison of the lung adenoma response in strain A/J mice after intraperitoneal and oral administration of carcinogens. *Toxicology and Applied Pharmacology*.

[87] Dow L. E., Lowe S. W. (2012). Life in the fast lane: mammalian disease models in the genomics era. *Cell*.

[88] Premsrirut P. K., Dow L. E., Kim S. Y. (2011). A rapid and scalable system for studying gene function in mice using conditional RNA interference. *Cell*.

[89] Zhou Y., Rideout W. M., Zi T. (2010). Chimeric mouse tumor models reveal differences in pathway activation between ERBB family- and KRAS-dependent lung adenocarcinomas. *Nature Biotechnology*.

[90] Kohl N. E., Omer C. A., Conner M. W. (1995). Inhibition of farnesyltransferase induces regression of mammary and salivary carcinomas in ras transgenic mice. *Nature Medicine*.

[91] Bos J. L. (1989). Ras oncogenes in human cancer: a review. *Cancer Research*.

[92] Lernert E. C., Qian Y., Hamilton A. D., Sebti S. M. (1995). Disruption of oncogenic K-Ras4B processing and signaling by a potent geranylgeranyltransferase I inhibitor. *Journal of Biological Chemistry*.

[93] Megaraj V., Zhou X., Xie F., Liu Z., Yang W., Ding X. (2014). Role of CYP2A13 in the bioactivation and lung tumorigenicity of the tobacco-specific lung procarcinogen 4-(methylnitrosamino)-1-(3-pyridyl)-1-butanone: in vivo studies using a CYP2A13-humanized mouse model. *Carcinogenesis*.

[94] Taguchi A., Politi K., Pitteri S. J. (2011). Lung cancer signatures in plasma based on proteome profiling of mouse tumor models. *Cancer Cell*.

[95] Perez-Soler R. (2004). The role of erlotinib (Tarceva, OSI 774) in the treatment of non-small cell lung cancer. *Clinical Cancer Research*.

[96] Eskens F. A. L. M., Mom C. H., Planting A. S. T. (2008). A phase I dose escalation study of BIBW 2992, an irreversible dual inhibitor of epidermal growth factor receptor 1 (EGFR) and 2 (HER2) tyrosine kinase in a 2-week on, 2-week off schedule in patients with advanced solid tumours. *British Journal of Cancer*.

[97] Natale R. B., Bodkin D., Govindan R. (2009). Vandetanib versus gefitinib in patients with advanced non-small-cell lung cancer: results from a two-part, double-blind, randomized phase II study. *Journal of Clinical Oncology*.

[98] Inoue S., Hartman A., Branch C. D. (2007). mda-7 in combination with bevacizumab treatment produces a synergistic and complete inhibitory effect on lung tumor xenograft. *Molecular Therapy*.

[99] Hanna N., Lilenbaum R., Ansari R. (2006). Phase II trial of cetuximab in patients with previously treated non-small-cell lung cancer. *Journal of Clinical Oncology*.

[100] Johnson D. H., Fehrenbacher L., Novotny W. F. (2004). Randomized phase II trial comparing bevacizumab plus carboplatin and paclitaxel with carboplatin and paclitaxel alone in previously untreated locally advanced or metastatic non-small-cell lung cancer. *Journal of Clinical Oncology*.

[101] Ichihara E., Ohashi K., Takigawa N. (2009). Effects of vandetanib on lung adenocarcinoma cells harboring epidermal growth factor receptor T790M mutation *in vivo*. *Cancer Research*.

[102] Wozniak A. J., Crowley J. J., Balcerzak S. P. (1998). Randomized trial comparing cisplatin with cisplatin plus vinorelbine in the treatment of advanced non-small-cell lung cancer: a Southwest Oncology Group study. *Journal of Clinical Oncology*.

[103] Raben D., Bianco C., Damiano V. (2004). Antitumor activity of ZD6126, a novel vascular-targeting agent, is enhanced when combined with ZD1839, an epidermal growth factor receptor tyrosine kinase inhibitor, and potentiates the effects of radiation in a human non-small cell lung cancer xenograft model. *Molecular Cancer Therapeutics*.

[104] Sordella R., Bell D. W., Haber D. A., Settleman J. (2004). Gefitinib-sensitizing EGFR mutations in lung cancer activate anti-apoptotic pathways. *Science*.

[105] Williams S. S., Alosco T. R., Mayhew E., Lasic D. D., Martin F. J., Bankert R. B. (1993). Arrest of human lung tumor xenograft growth in severe combined immunodeficient mice using doxorubicin encapsulated in sterically stabilized liposomes. *Cancer Research*.

[106] Abrams T. J., Lee L. B., Murray L. J., Pryer N. K., Cherrington J. M. (2003). SU11248 inhibits KIT and platelet-derived growth factor receptor *β* in preclinical models of human small cell lung cancer. *Molecular Cancer Therapeutics*.

[107] Rütters H., Zürbig P., Halter R., Borlak J. (2006). Towards a lung adenocarcinoma proteome map: studies with SP-C/c-raf transgenic mice. *Proteomics*.

[108] Cohen M. H., Williams G. A., Sridhara R. (2004). United States Food and Drug Administration drug approval summary: gefitinib (ZD1839; Iressa) tablets. *Clinical Cancer Research*.

[109] Thatcher N., Chang A., Parikh P. (2005). Gefitinib plus best supportive care in previously treated patients with refractory advanced non-small-cell lung cancer: results from a randomised, placebo-controlled, multicentre study (Iressa Survival Evaluation in Lung Cancer). *The Lancet*.

[110] Gerber D. E., Minna J. D. (2010). ALK inhibition for non-small cell lung cancer: from discovery to therapy in record time. *Cancer Cell*.

[111] Hallberg B., Palmer R. H. (2010). Crizotinib—latest champion in the cancer wars?. *The New England Journal of Medicine*.

[112] Wall M. E., Wani M. C. (1995). Camptothecin and taxol: discovery to clinic—thirteenth Bruce F. Cain Memorial Award Lecture. *Cancer Research*.

[113] Smit E. F., Mattson K., von Pawel J., Manegold C., Clarke S., Postmus P. E. (2003). ALIMTA (pemetrexed disodium) as second-line treatment of non-small-cell lung cancer: a phase II study. *Annals of Oncology*.

[114] Johansen P. B. (1981). Doxorubicin pharmacokinetics after intravenous and intraperitoneal administration in the nude mouse. *Cancer Chemotherapy and Pharmacology*.

[115] Oliver T. G., Mercer K. L., Sayles L. C. (2010). Chronic cisplatin treatment promotes enhanced damage repair and tumor progression in a mouse model of lung cancer. *Genes & Development*.

[116] Jensen P. B., Roed H., Skovsgaard T. (1990). Antitumor activity of the two epipodophyllotoxin derivatives VP-16 and VM-26 in preclinical systems: a comparison of *in vitro* and *in vivo* drug evaluation. *Cancer Chemotherapy and Pharmacology*.

[117] Ruckdeschel J. C. (1991). Etoposide in the management of non-small cell lung cancer. *Cancer*.

[118] Socinski M. A., Scappaticci F. A., Samant M., Kolb M. M., Kozloff M. F. (2010). Safety and efficacy of combining sunitinib with bevacizumab + paclitaxel/carboplatin in non-small cell lung cancer. *Journal of Thoracic Oncology*.

[119] Blumenschein G. R., Gatzemeier U., Fossella F. (2009). Phase II, multicenter, uncontrolled trial of single-agent sorafenib in patients with relapsed or refractory, advanced non-small-cell lung cancer. *Journal of Clinical Oncology*.

